# On the Difficulty of the Diagnosis between Pregnancy and Tumors of the Abdomen

**Published:** 1860-06

**Authors:** Robt. C. Croft

**Affiliations:** L. R. C. P., Edin., etc.


*On the Difficulty of Diagnosis between Pregnancy and Tumors of the Abdomen.* By ROBT. C. CROFT, Esq., L. R. C. P., Edin., etc.—There are few cases likely to prove more embarrassing, especially to the young practitioner, than those in which he is called to determine the nature of abdominal enlargements. These depend upon so many causes, and are so often masked, that it is frequently impossible to give an opinion with safety. When such protuberances exist in the male, the diagnosis, as far as pregnancy is concerned, is perfectly clear, but in the female, whenever her abdomen begins to enlarge, and if she be at the child-bearing age, pregnancy is at once suspected as the cause. There are two classes of women who come under our care who frequently baffle the medical man in his examination. These are unmarried women, who have hence no legal right to become mothers; and those who, being married, are eagerly desirous of becoming mothers. The first class will strongly deny symptoms that may exist, and place every obstacle in the way of the obstetrician; the second, by her hope of being pregnant, will be induced to describe imaginary symptoms. To illustrate these remarks he gives the following case:—

Mrs.——, aged forty-two, married nineteen years. No children or miscarriages; in bad health; has had fistulæ in ano. Six years ago was struck in the right lumbar region by the pole of an omnibus, from which she has suffered more or less ever since. Two years ago, injured again in the right groin. When requested to see her she believed herself *enceinte*, and was suffering from distressing sickness. Felt, six months previous to the visit of her physician, a peculiar sensation in the region of the uterus. The catamenia appeared regularly till two months ago, when they ceased entirely; the breasts were full and painful, and there was a tumor in the lower part of the abdomen.

The next visit she was in bed: her countenance pale and anxious, breasts full and tense, tender to the touch, containing milk, which could be extracted; nipples large and sore; areolæ raised and darkened; abdomen tense and tympanitic, and just above the symphysis pubis was felt a hard substance, the size of an orange, in the pelvis, and well defined, nearly in the middle line of the abdomen, and inclined to the *left* side; no discharge from the vagina. By a stethescopic examination a peculiar whirring sound was heard, like the placental murmur, to the *right* of the middle line above the pubis. From this time for three weeks no pain save in the breasts and nipples; the sickness constant; the only relief obtained was from ice. After a time the sickness abated, and food could be taken.

At her next menstrual period she suffered, for three days, violent uterine pains; no discharge, the tumor seemed to have increased in size, and to be higher in the abdomen.

She was seen by other practitioners, who seemed equally doubtful as to the existence of pregnancy. But slight changes were observed for some five weeks. Kiesteine, on every examination of the urine, was detected. Now a symptom arose; profuse spontaneous salivation came on. An examination, per vaginam, showed the cervix uteri shortened, the os opened, the lips puffy and swollen; on pressing upward with the finger, behind the symphysis, a hard substance was felt, very much resembling the head of a child at a late period of gestation. Dr. Robert Lee and Mr. Havers saw her, and while admitting the presence of most of the symptoms of pregnancy, yet declined to give a decided opinion. The only certain sign, the pulsation of the fœtal heart, could not be detected.

December 17th.—The condition of the patient pretty much as before; the tumor appearing to occupy the whole of the upper portion of the right half of the abdomen as high as the ribs, and extending deeply into the lumbar region. On pressure, a substance, like the gravid womb, was felt, and *in it* a distinct movement as of a fœtus. With the stethescope, the peculiar sound was heard as before, with a cooing as of a dove. Upon shifting the instrument a little higher up, the fœtal heart was heard distinctly. Being now certain of the existence of pregnancy, the tumor was attacked, by directing the nurse to rub it gently, night and morning, for a quarter of an hour, with liniment of iodide of potassium. By the first of the succeeding March, the patient was vomiting freely; the abdomen large, tense, dull on percussion; the umbilicus prominent; the ensiform cartilage pressed out; the abdomen measuring forty-two inches round. When a contraction of the uterus occurred, the tumor was very apparent.

May 10th.—After a severe labor, delivery took place. The placenta having been removed, a large firm mass was felt, seeming to occupy nearly the whole of the abdomen, and extending crosswise from the left ilium to the ribs on the opposite side. By firm pressure, the greater portion of the tumor sunk into the pelvis, leaving a large, round, hard ball loose in the abdomen. It was evident that the womb had been distended by the effusion into it of a quantity of blood. From this time all went well, the tumor remaining about the size of a large cricket ball, seemingly attached by a very small pedicle.—*Lancet*, January 28, 1860.

